# Antibiotic-Induced Gut Microbiota Dysbiosis Damages the Intestinal Barrier, Increasing Food Allergy in Adult Mice

**DOI:** 10.3390/nu13103315

**Published:** 2021-09-23

**Authors:** Qiuyu Zhang, Lei Cheng, Junjuan Wang, Mengzhen Hao, Huilian Che

**Affiliations:** Key Laboratory of Precision Nutrition and Food Quality, Key Laboratory of Functional Dairy, College of Food Science and Nutritional Engineering, China Agricultural University, Beijing 100083, China; SY20193061010@cau.edu.cn (Q.Z.); cl880416@163.com (L.C.); wangjunjuan93@163.com (J.W.); mengzhen.hao@cau.edu.cn (M.H.)

**Keywords:** food allergy, gut microbiota dysbiosis, antibiotic, intestinal barrier, tight junction proteins

## Abstract

(1) Background: The use of antibiotics affects the composition of gut microbiota. Studies have suggested that the colonization of gut microbiota in early life is related to later food allergies. Still, the relationship between altered intestinal microbiota in adulthood and food allergies is unclear. (2) Methods: We established three mouse models to analyze gut microbiota dysbiosis’ impact on the intestinal barrier and determine whether this effect can increase the susceptibility to and severity of food allergy in later life. (3) Results: The antibiotic-induced gut microbiota dysbiosis significantly reduced Lachnospiraceae, Muribaculaceae, and Ruminococcaceae, and increased Enterococcaceae and Clostridiales. At the same time, the metabolic abundance was changed, including decreased short-chain fatty acids and tryptophan, as well as enhanced purine. This change is related to food allergies. After gut microbiota dysbiosis, we sensitized the mice. The content of specific IgE and IgG1 in mice serum was significantly increased, and the inflammatory response was enhanced. The dysbiosis of gut microbiota caused the sensitized mice to have more severe allergic symptoms, ruptured intestinal villi, and a decrease in tight junction proteins (TJs) when re-exposed to the allergen. (4) Conclusions: Antibiotic-induced gut microbiota dysbiosis increases the susceptibility and severity of food allergies. This event may be due to the increased intestinal permeability caused by decreased intestinal tight junction proteins and the increased inflammatory response.

## 1. Introduction

The microbiota system, composed of resident gut microbiota, plays an essential role in intestinal barrier health. The imbalance in gut microbiota has been shown to be closely related to gastrointestinal inflammation, tumors, obesity, diabetes, and neuropsychiatric diseases [1,2,3,4]. Recently, scientists have focused on the relationship between intestinal flora and illness, especially food allergies [5,6].

Population analyses correlated the intestinal flora in early life with the occurrence and development of subsequent food allergies to explore the relationship between intestinal flora and allergies [7,8]. Savage JH et al. found that Clostridium, Dorea, Haemophilus, and Dialister were negatively correlated with food allergic sensitization in children at the age of three, and Oscillospira, Dorea, Lactococcus, and Citrobacter were negatively correlated with food allergies [9]. A small prospective study found that the abundance of intestinal microbiota was negatively associated with food sensitization. The ratio of Enterobacteriaceae/Bacteroides fragilis was positively correlated with food sensitization development at age one [10]. In a multicenter study, FazlollahiM et al. used 16SrRNA sequencing to characterize the intestinal microflora of 141 children with egg allergies for 3–16 months and a control group to explore the association between early intestinal flora and egg allergies. Three genera significantly associated with egg allergy were identified: Ruminococcus, Lactococcus, and Leuconostoc, of which Ruminococcus and Lactococcus were enriched in children with an egg allergy; the control group was rich in Leuconostoc. They also found that children with egg allergies and sensitivities had an increased diversity of their gut microbiome compared to subjects without egg allergies and sensitivities, which is inconsistent with some of the findings [8]. A study found that children who recovered from milk allergies at the age of eight had feces rich in Clostridium compared with children with persistent allergies. Therefore, the researchers used animal models to intervene, and the results confirmed the protective effect of Clostridium members on food sensitization [7]. Clostridium plays a protective role by inducing innate lymphocytes to produce IL-22, reducing intestinal barrier permeability and allergen exposure to the circulation [11].

Other studies have used animal models to examine the influence of intestinal flora colonization on allergic reactions [6,12]. Noval Rivas M found that allergic mice showed specific microbial characteristics, such as abundance changes of several bacterial families, including Lachnospiraceae, Lactobacillaceae, Rikenellaceae, and Porphyromonadaceae. Microflora transplantation and reconstruction in sterile mice promotes OVA-specific IgE responses and anaphylaxis [12]. These studies all show that the specific colonization of gut flora in early life can influence subsequent food allergies later in life. However, the specific mechanism is still unclear, and no definite and precise results can be obtained.

Many studies have shown that the gut microbiota dysbiosis is often accompanied by intestinal barrier damage, increasing intestinal permeability [13,14,15]. The intestinal barrier can effectively prevent toxins, antigens, and pathogens from penetrating into the circulatory system from the luminal environment [16]. Impairment of the intestinal barrier increases the risk of food allergies [17]. Intestinal epithelial cells (IECs) are closely arranged in the intestine, forming the main defense between the host and intestinal contents [18,19]. In the apical intercellular space of IECs, tight junction proteins (TJs) form a complex intercellular barrier and pores that regulate the paracellular passage of ions and molecules, and are essential for the integrity of the intestinal barrier [20,21]. Mast cell activation and the release of protease and inflammatory cytokines can affect TJ protein rearrangement and damage the intestinal barrier [22]. Secretory immunoglobulin A (sIgA) is the most abundant mucosal immunoglobulin isotype in the intestinal mucus layer. It is produced by specialized plasma cells found in lamina propria [23]. SIgA plays a vital role in the intestinal immune barrier. Besides this, goblet cells secreting mucin and glycoprotein are also essential for maintaining intestinal barrier health [18]. The gut flora itself is also an integral part of the intestinal barrier—the biological barrier. In healthy individuals, the gut microbiota comprises more than 1000 bacterial species that contribute to gastrointestinal homeostasis [24]. Resident intestinal bacteria promote the metabolism of nutrients, regulate immune homeostasis, and clear pathogens. An altered gut microbiota structure affects immune system development and function. Germ-free mice have reduced numbers of T helper cells (Th) 1 and Th17 cells, so intestinal T cell immune responses in germ-free animals are mainly controlled by Th2 cells [25]. Metabolites of gut flora also play an essential role in microbe–host interactions. Microbes benefit the host through the local synthesis of short-chain fatty acids (SCFAs), including butyrate, propionate, and acetate. They mainly enhance the intestinal barrier function of the host, including affecting the production of T regulatory cells, inducing mucosal B cells to produce IgA, and regulating the differentiation of Th17 cells [26]. Bacterial tryptophan metabolism produces effective bioactive metabolites, such as indole propionic acid (IPA), indole acrylic acid (IA), and indole acetic acid (IAA). These metabolites can activate the pregnane X receptor (PXR) and aromatic hydrocarbon receptor (AHR), affecting the integrity of the intestinal barrier and host immunity [19,27,28,29]. These findings suggest that the phenomenon of gut microbiota dysbiosis promoting food allergies may be related to the damage of the intestinal barrier. Still, more evidence is needed to explain the specific mechanism.

A study found that the antibiotic treatment of newborns reduced microbial diversity and bacterial load in stool and ileum samples, and increased sensitization to food allergens. Even low doses of early antibiotic exposure can have long-term effects on metabolism and immune responsiveness [30]. A large population-based follow-up study found that the use of antibiotics in the first few months of a baby’s life was associated with an increased risk of milk allergy [31]. Furthermore, these results suggest that the use of antibiotics in early life can increase the risk and severity of food allergies in later life. However, it is still unknown whether a similar association between intestinal flora and food allergy exists in adulthood. Whether the disturbance of intestinal flora can be an indicator that helps to predict the occurrence of food allergies has also become a question worth exploring.

Therefore, first of all, our study established a mice model of gut microbiota dysbiosis to observe whether the composition of intestinal flora and changes in metabolites may be correlated with food allergies. Next, we sensitized mice with gut microbiota dysbiosis by gavage OVA to observe whether gut microbiota dysbiosis can increase food allergy susceptibility. Finally, after sensation, the animals were given antibiotics to induce intestinal flora disorders. We assessed whether gut microbiota dysbiosis can aggravate food allergies, and explored the reasons for this phenomenon. This study sought to determine whether antibiotics aggravate food allergies, and further explores the relationship between intestinal flora disorders and food allergies.

## 2. Materials and Methods

### 2.1. Materials

Metronidazole, ampicillin, neomycin sulfate, and vancomycin were obtained from Macklin Biochemical Technology (Shanghai, China). Mouse anti-DNP-IgE monoclonal antibody, DNP-HSA, ovalbumin (OVA), cholera toxin (CT), and aluminum were obtained from Sigma-Aldrich. Mouse IL-4, IL-5, IFN-γ, TNF-α and mMCP-1, histamine, zonluin, and the sIgA ELISA kit were from Dongge Boye (Beijing, China). Mouse ZO-1, Claudin-1, and Occludin polyclonal antibodies were obtained from Lianke Biotechnology (Hangzhou, China). The FastDNA SPIN Kit for soil was from Mpbio (Santa Ana, CA, USA). PicoGrenn dsDNA was from Life Technology. The AXYPREP DNA Gel Extraction Kit was obtained from Axygen Biosciences. The Roche GS FLX Titanium em PCR Kit and Roche GS FLX Sequencing kit were from Roche Applied Science. DNA DL2000Marker was obtained from Takara Bio (Dalian, China).

### 2.2. Animal Models

The female BALB/c mice (6 weeks old) weighing 18–20 g used in our research were purchased from Vital River Laboratories, Inc. (Beijing, China) and housed in the specific pathogen-free (SPF) animal laboratory of the College of Food Science and Nutritional Engineering, China Agricultural University (Beijing, China). Animal rooms were maintained at a temperature of 22 ± 1 °C, a humidity of 55 ± 5%, 12 h light/dark cycles, and air exchanges at 15 times/h. We applied adaptive feeding for a week, with free food and water intake. All animal experiments were performed under the China Agricultural University Animal Experimental Welfare and Ethical Inspection Committee-approved protocols and under the ethical standard guidelines of China Agricultural University. All efforts were made to minimize the suffering of the experimental animals.

#### 2.2.1. Gut Microbiota Dysbiosis Model

**Figure 1:**
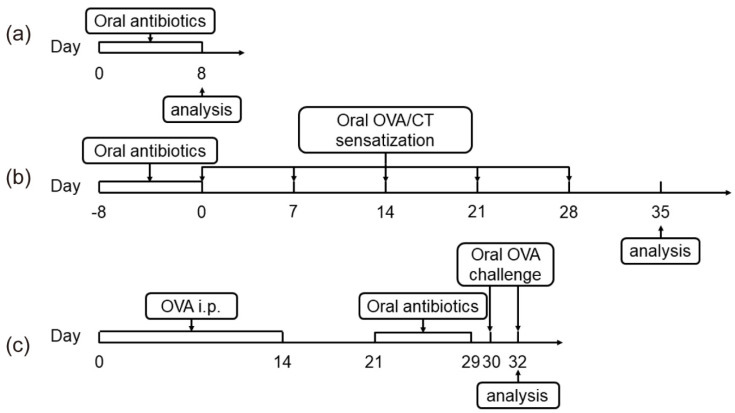
Methods of animal model establishment (**a**). Gut microbiota dysbiosis model (**b**). Gut microbiota dysbiosis-food sensitization model (**c**). Food allergy-gut microbiota dysbiosis model.

Thirty female BALB/c mice were divided into two groups (*n* = 15) with equal bodyweights after a week of acclimation. The control group (Ctrl) was given sterile water without antibiotics, while the antibiotic treatment group (Intes) was given sterile water with metronidazole (1 g/L), ampicillin (1 g/L), neomycin sulfate (1 g/L), and vancomycin (500 mg/L). Simultaneously, the antibiotic group was given 0.2 mL of 300 mg/mL ceftriaxone sodium solution once at 10 am every day, while the Ctrl group was given 0.2 mL phosphate buffer solution (PBS) simultaneously. The behavior, coat luster, mental state, and feces morphology of the mice were observed every day. On the 8th day of the experiment, the mouse feces were was and not formed, and antibiotics were stopped. The specific method of drug delivery is shown in Figure 1a.

#### 2.2.2. Gut Microbiota Dysbiosis—Food Sensitization Model

In total, 70 female BALB/c mice were divided into two groups (normal group = 40, gut microbiota dysbiosis group = 30) with equal bodyweights after a week of acclimation. During the eight days before the experiment, the treatment was the same as in the gut microbiota dysbiosis model. Antibiotics were discontinued on day 0. The mice were divided into groups again and sensitized by gavage OVA and CT. Forty mice in the normal group were divided into four groups (*n* = 10): OVA-CT-Antibiotic-, OVA-CT+Antibiotic-, OVA+CT-Antibiotic-, and OVA+CT+Antibiotic-. Thirty mice in the gut microbiota dysbiosis group were divided into three groups (*n* = 10): OVA-CT-Antibiotic+, OVA+CT-Antibiotic+, and OVA+CT+Antibiotic+. The OVA+CT+Antibiotic- and OVA+CT+Antibiotic+ groups were sensitized by gavage of a 200 μL OVA solution (1 mg OVA and 10 μg CT in 0.9% NaCl) on days 0, 7, 14, 21, 28, and 35. Other groups were treated with normal saline, CT, and OVA at the same time. The specific method of drug delivery is shown in Figure 1b.

#### 2.2.3. Food Allergy—Gut Microbiota Dysbiosis Model

Forty female BALB/c mice were divided into two groups (control group = 20, sensitization group = 20) with equal bodyweights after a week of acclimation. Mice in the sensitization group were intraperitoneally injected with a 100 μL OVA solution (200 μg OVA and 20 μL aluminum in PBS). The mice in the control group were injected with the same dose of PBS. On day 21, we detected the serum-specific IgE and IgG1 levels of mice to determine sensitization success, and then the mice were divided into groups again. Twenty mice in the control group were divided into two groups (*n* = 10): OVA-Antibiotic- and OVA-Antibiotic+. Twenty mice in the sensitization group were divided into two groups (*n* = 10): OVA+Antibiotic- and OVA-Antibiotic+. During days 21–29, we treated the mice in the OVA-Antibiotic+ group and the OVA+Antibiotic+ group with antibiotics according to the method in the gut microbiota dysbiosis model, and the other two groups were given PBS at the same time. In total, 50 mg OVA was provided by gavage at 30 d and 32 d, and allergy-related indexes were observed and recorded. The specific method of drug delivery is shown in Figure 1c.

### 2.3. Preparation of Total DNA and Illumina High-Throughput Sequencing Analysis

Total DNA was extracted from the mice cecum according to the instructions of the FastDNA SPIN kit, and then measured using a NanoDrop ND-2000 Spectrophotometer (Thermo Fisher Scientific, Wilmington, NC, USA) and 1% agarose gel electrophoresis. The V3–V4 region of the 16S rRNA gene was selected for PCR amplification using a forward primer (5′-ACTCCTACGGGAGGCAGCAG-3′) and a reverse primer (5′-GGACTACHVGGGTWTCTAAT-3′). The PCR products were recovered and purified, and then quantified by QuantiFluor™-ST. The DNA library was constructed, then subsequently sequenced using the IlluminaMiSeq PE300 System.

### 2.4. Bioinformatics Analysis

We used Trimmomatic software for the quality control of the original sequencing sequences. We used FLASH software to stitch together a sequence. UPARSE was used to perform OTU clustering of the sequences with 97% similarity as the standard, and single sequences and chimeras were removed during the clustering process. The RDP classifier was used to annotate the species of each sequence, and the threshold was set to 70% for comparison with the Silva database (SSU123). Phylogenetic Investigation of Communities by Reconstruction of Unobserved States (PICRUSt) (http://picrust.github.com) (accessed on 13 May 2019) software was used to predict the functions of the KEGG categories represented in mice.

### 2.5. Clinical Symptom Score

After high-dose allergen stimulation, the mice were observed for 45 min. Allergic symptoms were scored according to the behavior of the mice. The scoring standards, according to Yao et al., were as follows: 0, no obvious symptoms; 1, scratch nose, scratch head.; 2, puffiness around the eyes and mouth, erect hair, reduced activity or shortness of breath; 3, difficulty breathing, pale around the mouth and tail; 4, convulsion or stillness after stimulation; 5, death [32].

### 2.6. ELISA

Blood was collected from the canthal vein of mice in a 1.5 mL centrifuge tube, then centrifuged at 3000 rpm and 4 °C for 10 min. The supernatant was collected and stored at −80 °C. Serum samples were used to quantify OVA-specific IgE, IgG1, histamine, cytokines (IL-4, IL-5, IFN-γ), mMCP-1, and zonulin using commercial mouse ELISA kits (Beijing, China).

About 3 cm of jejunum tissue was collected and put into an aseptic cryopreservation tube. The jejunum samples were used to quantified sIgA using commercial mouse ELISA kits (Beijing, China).

### 2.7. Histopathological Observation

About 3 cm samples of the jejunum and colons of mice were collected and fixed with 4% formaldehyde solution. The jejunum and colon were stained with hematoxylin-eosin (HE) for histopathological observation.

### 2.8. Goblet Cell Staining

The jejunal tissue sections were dewaxed with water and then stained in Periodic Acid–Schiff (PAS) solution. The sections were observed with a microscope. Quantitative analysis was carried out via the Image-Proplus6.0 system.

### 2.9. Immunohistochemical Staining

The jejunum of mice was fixed with 4% formaldehyde solution. The jejunal tissue sections with a thickness of 4 μ m were fixed on a slide coated with poly-lysine. Paraffin wax was dewaxed and incubated with 3% hydrogen peroxide for 10 min. The sections were incubated with the closed serum at room temperature for 30 min, and then the anti-ZO-1 antibody, anti-occludin antibody, and anti-claudin-1 antibody were used overnight at 4 °C. After incubation with a biotinylated second antibody, the slices were incubated in horseradish peroxidase avidin–biotin complex reagent for 30 min. After that, the sections were stained with 3,3′-diaminobenzidine (DAB). The Image Proplus 6.0 system was used for the quantitative analysis.

### 2.10. Statistical Analysis

All data were presented as the mean values ± standard error of mean (SEM) from three independent biological replicates. Statistical significance was determined by one-way analysis of variance (ANOVA) using the SPSS Statistics 25 software. Differences were considered significant at *p* < 0.05.

## 3. Results

### 3.1. Antibiotics Reduce the Diversity of Intestinal Flora

**Figure 2:**
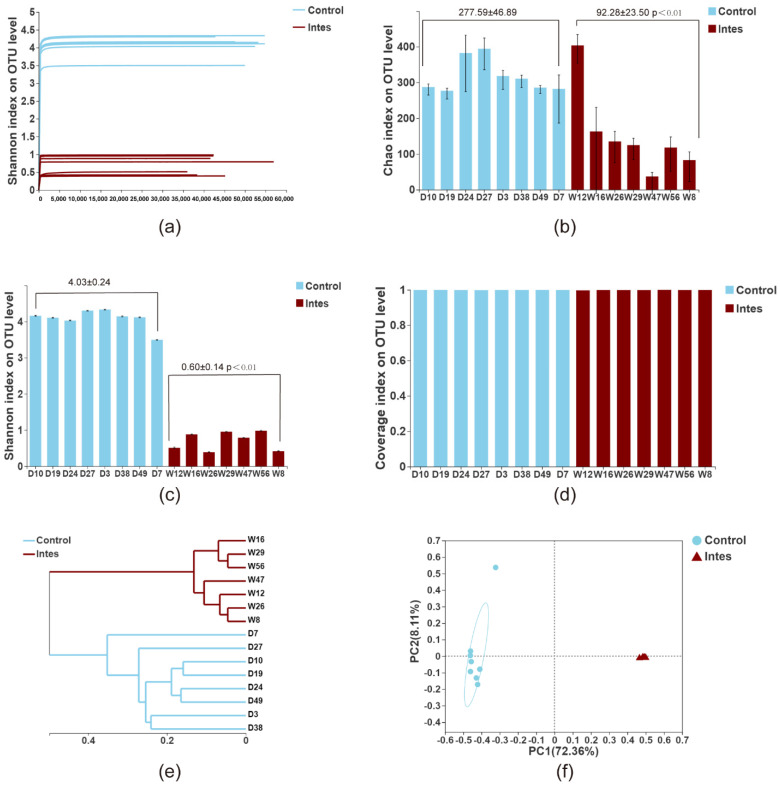
Analysis of α and β diversity of intestinal flora (**a**). Shannon-Wiener diversity curve (**b**). Chao index (**c**). Shannon index (**d**). Coverage index (**e**). Sample hierarchical clustering (**f**). PCoA. *p* < 0.01 as compared to the Ctrl group.

We used the IlluminaMiSeq to perform high-throughput sequencing of the cecal contents of 15 mice in the two groups of the gut microbiota dysbiosis model and obtained 775,800 optimized and effective sequences. In the Shannon–Wiener diversity curve (Figure 2a), each sample’s curve rises to a flat level, indicating that the sequence is sufficient to cover most of the microorganisms in the sample. The Chao index (Figure 2b) reflects the microbial community richness in this experiment, while the Shannon index (Figure 2c) reflects the species diversity [33]. We found that antibiotic intervention significantly reduced the richness and diversity of intestinal microflora compared to the Ctrl group. We also found no significant difference in the coverage index (Figure 2d) of species between the two groups. The sequencing coverage of all samples was higher than 0.999, indicating that the sequencing depth was deep enough to cover most microorganisms, including rare species.

We used hierarchical clustering to display the sample similarity through a visual tree (Figure 2e). The results showed that the intestinal flora colonization levels of the Ctrl group and the antibiotic group were significantly different. Principal coordinates analysis (PCoA) based on unweighted Unifrac assessed the differences between the groups (Figure 2f). The results show that the Ctrl group and the antibiotic group could be distinguished significantly along the PC1 axis. In this experiment, antibiotic treatment significantly changed the intestinal flora of mice.

### 3.2. Gut Microbiota Dysbiosis Shows Correlation with Food Allergy

To understand whether the antibiotic-mediated gut microbiota dysbiosis may be related to food allergy, we focused on the composition of the intestinal flora in the gut microbiota dysbiosis model mice through high-throughput sequencing, and the metabolism of the intestinal flora was predicted.

#### 3.2.1. Intestinal Flora Composition

**Figure 3:**
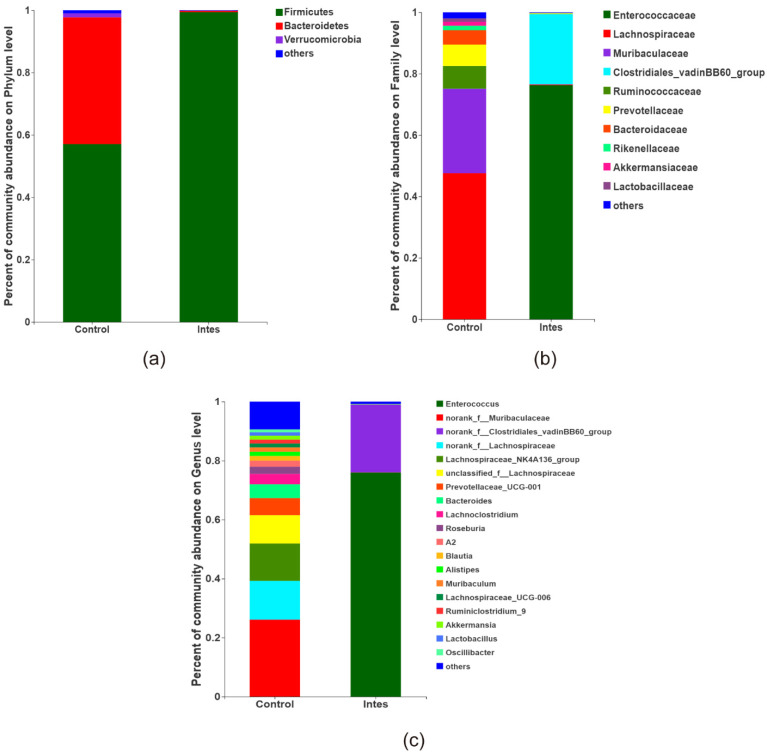
The composition of the intestinal microflora (**a**). Phylum level (**b**). Family level (**c**). Genus level.

Firmicutes and Bacteroidetes are the main components of human intestinal flora, accounting for more than 80% of the total flora, followed by Verrucomicrobia [34,35]. The phylum level of the intestinal flora is shown in Figure 3a. The results show that Firmicutes and Bacteroidetes accounted for more than 90%, and were the dominant component in the intestinal flora of control mice, followed by Verrucomicrobia, Actinobacteria, and Proteobacteria. The use of antibiotics significantly reduced the level of Bacteroidetes, and the dominant bacteria group was Firmicutes. The effect of the antibiotics used in our study on the colonization of intestinal flora is similar to that of obesity [36]. Some studies have pointed out that the colonization of intestinal flora in obese children is related to food allergies [6]. Compared with healthy children, the number of Bacteroides in children with food allergies was significantly reduced, and the number of Firmicutes increased significantly [5,37]. The antibiotics used in this study led to a significant increase in the relative abundance of Firmicutes and a considerable decrease in the relative abundance of the Bacteroides phylum (*p* < 0.05), causing disturbances in the intestinal flora. The two main bacterial phyla that changed were related to food allergies.

The family level of the intestinal flora is shown in Figure 3b. The dominant bacteria families in the Ctrl group were Lachnospiraceae (47.90%), Muribaculaceae (27.21%), Ruminococcaceae (7.28%), and Prevotellaceae (7.05%). After antibiotic treatment, the bacteria species in the gut microbiota dysbiosis group decreased, and the main dominant bacteria were Enterococcaceae (76.80%) and Clostridiales (22.21%). Antibiotic treatment significantly reduced Lachnospiraceae, Muribaculaceae, and Ruminococcaceae, while increasing the levels of Enterococcaceae and Clostridiales (*p* < 0.05). Previous studies have shown that Ruminococcaceae can produce beneficial short-chain fatty acids, such as butyrate and propionate. The short-chain fatty acids have important metabolic effects, inhibit lipopolysaccharide synthesis, reduce intestinal permeability, reduce inflammation and metabolic endotoxemia, and play a protective role against food allergy [10,38].

The genus level of the intestinal flora is shown in Figure 3c. The main dominant bacterial genera in the Ctrl group were norank_f__Muribaculaceae, Lachnospiraceae_NK4A136_group, norank_f__Lachnospiraceae, unclassified_f__Lachnospiraceae, Prevotellaceae_UCG-001, Bacteroides, etc. These genera were significantly reduced in the antibiotic group (*p* < 0.05). On the contrary, the dominant bacteria in the gut microbiota dysbiosis group were Enterococcus and norank_f__Clostridiales_vadinBB60_.

#### 3.2.2. Intestinal Flora Metabolites

**Figure 4:**
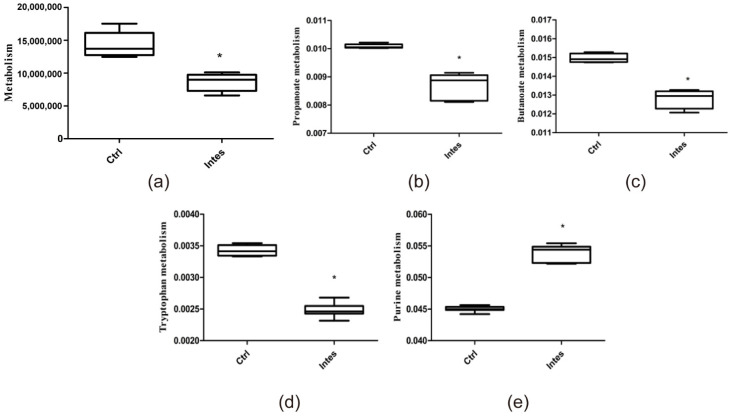
Changes in the metabolic abundance of mice in each group (**a**). KEGG pathway level 1 (**b**). SCFAs: propionate (**c**). SCFAs: butyrate (**d**); tryptophan (**e**); purine. * *p* < 0.05 as compared to the Ctrl group.

Some small molecules produced by the intestinal microbiome may influence the host’s health [39,40], so we predict the metabolic function of the intestinal microflora. We found that the abundance of metabolites in the gut microbiota dysbiosis group was reduced (Figure 4a) (*p* < 0.05), indicating that the antibiotics used in this study significantly affected the intestinal flora’s metabolic pathways.

Intestinal flora can ferment and metabolize dietary fiber to produce short-chain fatty acids (SCFAs), mainly butyrate, propionate, and acetate. Sufficient research has shown that SCFAs can affect the intestinal barrier’s health, such as affecting the production of Treg cells, inducing mucosal B cells to produce IgA, and regulating the differentiation of Th17 cells. SCFAs are also related to the risk of allergy [41]. As shown in Figure 4b,c, we found that the abundance of propionate and butyrate metabolic pathways was significantly reduced in the gut microbiota dysbiosis group (*p* < 0.05). The antibiotics selected in this study affect the intestinal flora of mice, which may affect the level of SCFAs, and then affect the intestinal barrier’s health.

Tryptophan is the only amino acid that contains indoles. Intestinal flora can produce indoles and indoles derivatives, such as indole propionic acid (IPA), indoleacetic acid (IA), and indoleacetic acid (IAA), through tryptophan metabolism. Studies have shown that tryptophan metabolites are related to intestinal barrier function: reduced IPA levels can increase intestinal barrier permeability [19]; IA promotes goblet cell differentiation and mucus secretion to enhance intestinal barrier function [42]. As shown in Figure 4d, antibiotics significantly reduce the metabolic abundance of tryptophan (*p* < 0.05). Gut microbiota dysbiosis may affect tryptophan metabolism, which in turn affects the health of the intestinal barrier.

At the same time, we found that gut microbiota dysbiosis caused significant abnormalities in purine metabolism (Figure 4e) (*p* < 0.05). The purine metabolism of the intestinal flora may affect tissue uric acid (UA) levels and inflammation [43]. UA can directly interact with the membrane of dendritic cells (DCs) and activate DCs. UA was significantly increased in children with peanut allergies [44]. UA is associated with the occurrence of allergic diseases and is an “alarm” molecule for allergies. Abnormal purine metabolism caused by gut microbiota dysbiosis may affect the occurrence of food allergies.

### 3.3. Increased Susceptibility to Food Allergy in Mice with Gut Microbiota Dysbiosis

**Figure 5:**
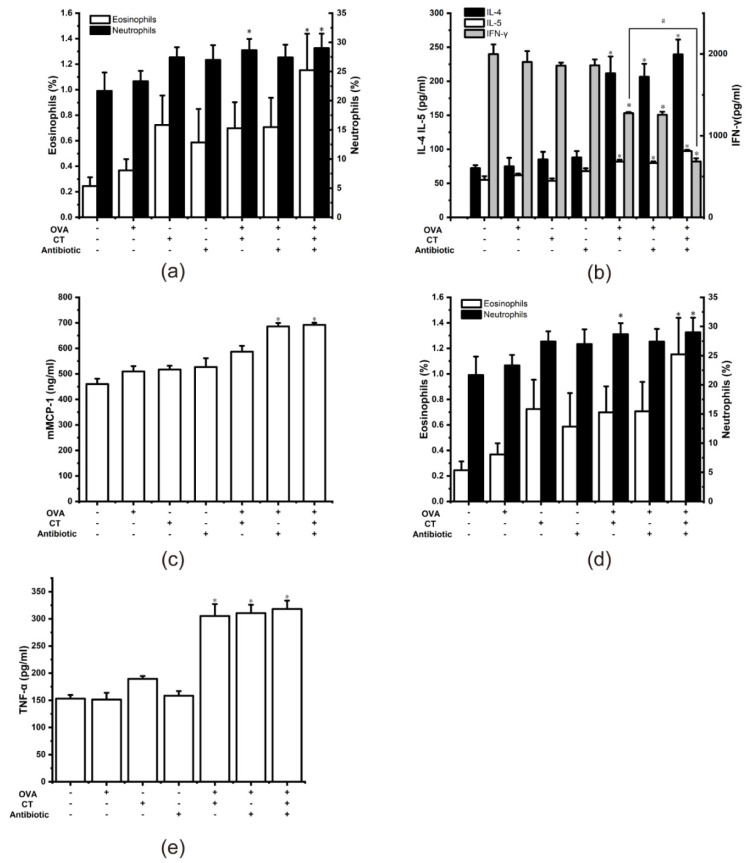
Increased susceptibility to food allergy in mice with gut microbiota dysbiosis (**a**). Specific antibody (**b**). Th1/Th2 cytokines (**c**). mMCP-1 (**d**). Percentage of eosinophils and neutrophils (**e**). TNF-α. * *p* < 0.05 as compared to the OVA-CT-Antibiotic- group, # *p* < 0.05 as compared to the OVA+CT+Antibiotic+ group.

After eight days of antibiotic treatment in mice, we stopped using antibiotics and gavage OVA for sensitization to explore whether gut microbiota dysbiosis would affect food allergy sensitization. Mouse serum was collected on the 35th day, and the OVA-specific IgE and IgG1 were determined. As shown in Figure 5a, the serum-specific IgE and IgG1 of the OVA+CT+Antibiotic- group both increased (*p*<0.05). Compared with the OVA-CT-Antibiotic- group, the specific IgE and IgG1 of the OVA+CT-Antibiotic+ group also increased significantly (*p* < 0.05). There was no significant difference between the OVA+CT+Antibiotic- group and the OVA+CT-Antibiotic+ group. The above results mean that only gavage OVA after gut microbiota dysbiosis can successfully sensitize mice. In other words, intestinal flora disorder can play a role similar to the CT adjuvant, promote the sensitization of mice, and increase food allergy susceptibility.

The differentiation of Th2 type cells is a critical link that affects food allergy sensitization. We measured the serum cytokines IL-4, IL-5 and IFNγ to explore the differentiation of Th cells. As shown in Figure 5b, Th2 cytokines IL-4 and IL-5 of the OVA+CT-Antibiotic+ group were significantly increased compared to the OVA-CT-Antibiotic- group (*p* < 0.05), while the Th1 cytokine IFN-γ was reduced (*p* < 0.05). Compared with the OVA+CT+Antibiotic- group, the IFN-γ levels of the OVA+CT+Antibiotic+ group were also lower (*p* < 0.05). We found that gut microbiota dysbiosis disrupted the Th1/Th2 balance in the sensitized mice and promoted the shift of the balance towards the Th2 type, which in turn led to the increased susceptibility of mice to food allergy.

In food allergies, mast cells are activated to secrete histamine and release mMCP-1. As shown in Figure 5c, serum MMCP-1 was significantly increased in the OVA+CT-Antibiotic+ group compared with the OVA-CT-Antibiotic- group (*p* < 0.05). The mice in the OVA+CT+Antibiotic- group were sensitized successfully, but there was no significant increase in serum mMCP-1 levels. These results suggest that gut microbiota dysbiosis increases susceptibility to food allergies, possibly due to its effect on mast cell status.

### 3.4. Inflammatory Response

Damage to the intestinal barrier caused by gut microbiota dysbiosis may lead to higher levels of inflammation, the proliferation of immune cells in the blood, and elevated cytokines associated with inflammation in the serum. TNF-α plays a crucial role in the inflammatory response and is the earliest and most vital mediator in the inflammatory response [45]. We collected mouse whole blood on the 35th day to detect the percentage changes of eosinophils, basophils, neutrophils, and lymphocytes, and detect the TNF-α in the serum of mice. As shown in Figure 5d, the ratio of eosinophils to neutrophils in the OVA+CT+Antibiotic+ group was significantly increased compared to the OVA-CT-Antibiotic- group (*p* < 0.05). In contrast, the OVA+CT-Antibiotic+ group showed no significant change, indicating that gut microbiota dysbiosis has no significant effect on the proliferation of blood immune cells. Compared with the OVA-CT-Antibiotic- group, the level of TNF-α in the OVA+CT-Antibiotic+ group was significantly increased (Figure 5e) (*p* < 0.05), but there was no significant difference compared with the OVA+CT+Antibiotic+ group. Gut microbiota dysbiosis participates in the immune response of food allergies and promotes the occurrence of TNF-α-mediated systemic inflammation, which may be related to an increased susceptibility to food allergies. It is worth noting that the TNF-α in the OVA-CT-Antibiotic+ group was increased compared with the OVA-CT-Antibiotic- group, but it was not significant (*p* < 0.05). This may be due to the long experimental period, which partially restored the inflammatory state caused by gut microbiota dysbiosis.

### 3.5. Intraperitoneal Injection of OVA Produces Systemic Allergy

**Figure 6:**
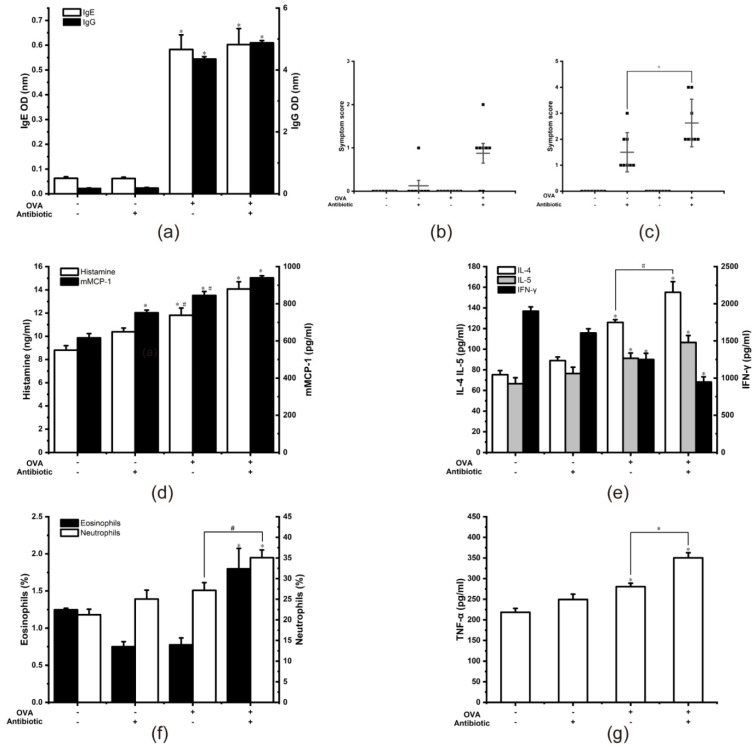
Gut microbiota dysbiosis causes more severe symptoms when mice that are already in a sensitized state are re-exposed to allergens (**a**). Specific IgE and IgG1 (**b**). Allergy symptom score of the first challenge (**c**). Allergy symptom score of the second challenge (**d**). Histamine and mMCP-1 (**e**). Th1/Th2 cytokine (**f**). Blood cells, including the percentage of eosinophils and neutrophils (**g**). TNF-α. * *p* < 0.05 as compared to the OVA-Antibiotic- group. # *p* < 0.05 as compared to the OVA+Antibiotic- group.

To understand whether antibiotic-mediated gut microbiota dysbiosis can increase the severity of food allergies, we established a food allergy–gut microbiota dysbiosis model. OVA-sensitized mice were injected intraperitoneally. We determined OVA-specific IgE and IgG1 on day 21. As shown in Figure 6a, the IgE and IgG1 in the OVA+Antibiotic- group and the OVA+Antibiotic+ group were significantly increased compared with the Ctrl group, indicating that the mice were in the sensitization state (*p* < 0.05).

### 3.6. Increased Severity of Food Allergies

After sensitization, the mice were given an antibiotic treatment to induce gut microbiota dysbiosis. Finally, the mice were given a high-dose OVA challenge twice, and the allergic symptoms were scored. We found that after the first challenge, the mice in the OVA+Antibiotic+ group already showed allergic symptoms (Figure 6b). Five mice showed typical nasal scratching behavior, and one mouse showed reduced activity. The OVA+Antibiotic- group mice did not show allergic symptoms, and only one mouse showed a slight nasal scratching behavior. After the second challenge, the allergy symptom score was also assessed (Figure 6c). The symptom score of the OVA+Antibiotic+ group was higher than the OVA+Antibiotic- group. Two mice had convulsions, twitching, or resting behavior after stimulation, and most of them had reduced activity, but the OVA+Antibiotic+ group mainly showed nasal scratching behavior. The above phenomena show that gut microbiota dysbiosis aggravates the symptoms of food allergy.

After the second challenge, we measured serum histamine and mMCP-1 levels (Figure 6d). The serum histamine in the OVA+Antibiotic+ group was significantly higher than that in the OVA+Antibiotic- group. Similar to histamine, compared with the OVA-Antibiotic- group, the OVA+Antibiotic+ group, and the OVA+Antibiotic- group, serum mMCP-1 increased significantly (*p* < 0.05). A food allergy challenge after gut microbiota dysbiosis may affect mast cells’ activity, thereby increasing the severity of food allergy.

Similarly, we measured the serum cytokines IL-4, IL-5 and IFN-γ to explore the differentiation of Th cells. IL-4 plays an essential role in the early stage of Th2 cell differentiation, and is the key to triggering IgE class switching [46]. We found that, compared with the OVA-Antibiotic- group, IL-4 was significantly increased in the OVA+Antibiotic+ group and the OVA+Antibiotic- group, and there was a significant difference between the two groups (Figure 6e). IL-5 promotes the accumulation of eosinophils in the gut in food allergies and exacerbates eosinophil-mediated inflammatory responses [47]. We found similar results with IL-4. Compared with the OVA-Antibiotic- group, IL-5 was significantly increased in the OVA+Antibiotic+ group and the OVA+Antibiotic- group. However, there was no significant difference between the two groups. The levels of typical Th1 cytokine IFN-γ were lower in the OVA+Antibiotic+ group and the OVA+Antibiotic- group compared to the OVA-Antibiotic- group. The above results indicate that gut microbiota dysbiosis might disrupt Th1/Th2 balance in allergic mice, promote the balance shift to Th2 type, and lead to the aggravation of food allergy symptoms.

To assess systemic inflammation in mice, we collected whole blood on day 32 and measured eosinophils, basophils, neutrophils, and lymphocyte percentage, as well as TNF-α. Compared with the OVA-Antibiotic- group, eosinophils and neutrophils were significantly increased in the OVA+Antibiotic+ group (Figure 6f). The levels of neutrophils were also markedly higher here than those in the OVA+Antibiotic- group. TNF-α levels in the OVA+Antibiotic+ group and the OVA+Antibiotic- group were higher than in the OVA-Antibiotic- group, and there was also a significant difference between the two groups (Figure 6g). This indicates that the antibiotic-treated mice’s blood immune cells proliferated, and the pro-inflammatory cytokines also increased, resulting in more serious systemic inflammation.

### 3.7. Intestinal Barrier Injury

**Figure 7:**
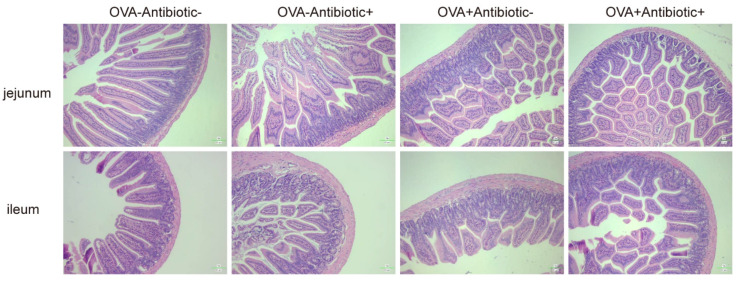
HE staining of sections of jejunum and ileum in the food allergy–gut microbiota dysbiosis model.

We observed mouse intestinal barrier changes via hematoxylin-eosin staining in the food allergy–gut microbiota dysbiosis model (Figure 7). The ileum and colon in mice of the OVA-Antibiotic- group exhibited normal histological features and displayed a complete villous structure. However, the intestinal tissues of mice in the other groups were damaged. In the OVA-Antibiotic+ group, the jejunum villi were slightly shed, while the ileum villi were seriously shed, with mucosal erosion and inflammatory cellsinfiltration. On the contrary, mice in the OVA+Antibiotic- group showed more severe jejunal injury, with the intestinal mucosal epithelial cells atrophied and desquamated. Compared to the OVA+Antibiotic- group, mice’s intestinal tissues in the OVA+Antibiotic+ group were more severely damaged. This result suggests that the dysbiosis of gut flora caused by antibiotic treatment results in intestinal pathological changes. Its influence on the severity of food allergies may result from its damaging of the intestinal barrier.

**Figure 8:**
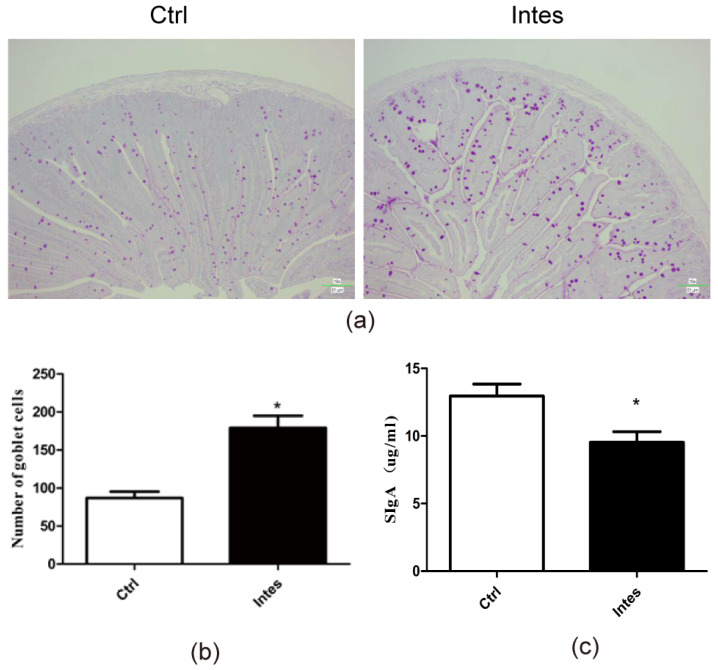
Mice with intestinal flora disorders showed more severe intestinal barrier damage (**a**). PAS staining of jejunum goblet cells in the gut microbiota dysbiosis model (**b**). Column graphs represent quantified goblet cell content (**c**). Jejunum sIgA content in the gut microbiota dysbiosis model. * *p* < 0.05 as compared to the Ctrl group.

Among intestinal epithelial cells, goblet cells enhance intestinal homeostasis and tolerance [48]. Therefore, we examined the contents of jejunum goblet cells in mice in the gut microbiota dysbiosis model. As shown in Figure 8a,b, we found that the levels of goblet cells in the antibiotic group increased significantly compared to the control (*p* < 0.05). This result shows that the dysbiosis of intestinal flora affects the state of goblet cells.

In the intestinal barrier, sIgA has a protective effect on the mucosa [49]. Intestinal mucosal damage often leads to reduced SIgA secretion. As such, we analyzed the changes in intestinal sIgA secretion in mice. As shown in Figure 8c, intestinal flora dysbiosis significantly reduced the sIgA content in mice’s jejunum (*p* < 0.05). This result indicates that the dysbiosis of the intestinal flora damages the intestinal barrier of mice, reduces the ability to block allergens from entering the body, and may promote food allergies.

### 3.8. The Decrease in Tight Junction Proteins and the Increase in Zonulin

**Figure 9:**
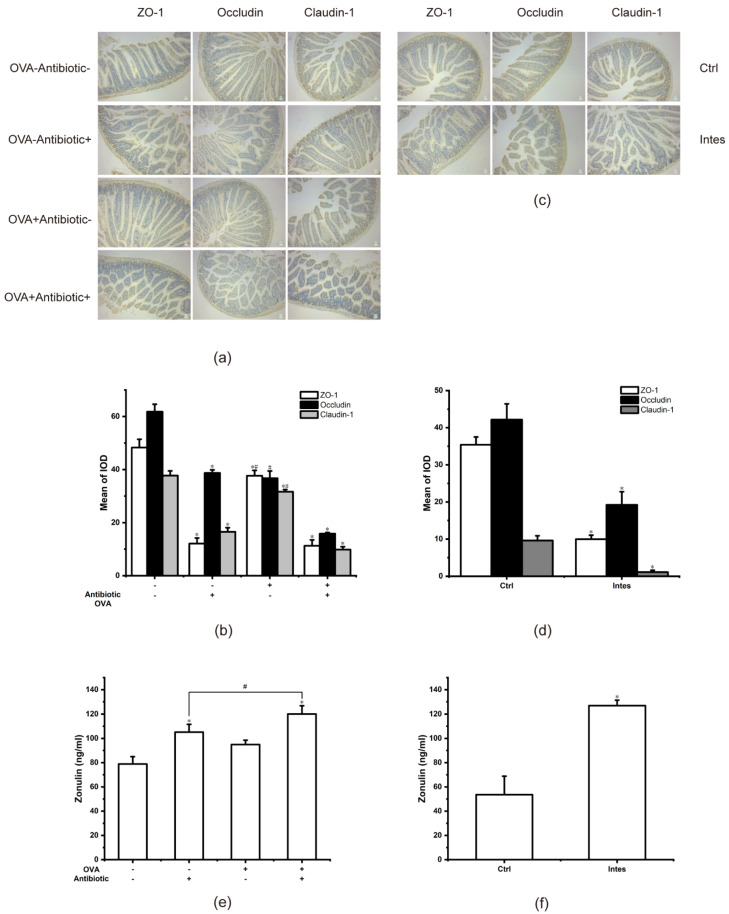
Dysbiosis of gut microbiota reduces tight junction proteins in jejunum tissues and increases zonulin in mice serum. (**a**) Immunohistochemical pictures and quantitative analysis of TJs in the food allergy–gut microbiota dysbiosis model. (**b**) Column graphs represent the quantitative result of (**a**). (**c**) Immunohistochemical pictures and quantitative analysis of TJs in the gut microbiota dysbiosis model. (**d**) Column graphs represent the quantitative result of (**c**). (**e**) Zonulin content in mice serum in the food allergy–gut microbiota dysbiosis model. (**f**) Zonulin content in mice serum in the gut microbiota dysbiosis model. * *p* < 0.05 as compared to the OVA-Antibiotic- group. # *p* < 0.05 as compared to the OVA+Antibiotic+ group.

Tight junction proteins (TJs) are the most important intercellular connection entities, and their absence and variation will lead to an increase in gut permeability and the destruction of intestinal homeostasis. As such, we focused on the effect of gut microbiota dysbiosis on TJs (Figure 9a,b). In the OVA-Antibiotic- group, the TJs, including ZO-1, Claudin-1, and Occludin, were positively expressed on the jejunal epithelium membrane in mice, showing brown–yellow colorations. However, the brown–yellow colorations of ZO-1 in both the OVA+Antibiotic+ group and the OVA+Antibiotic- group were significantly reduced (*p* < 0.05), indicating a reduced expression of the ZO-1 protein on the intestinal epithelial cells. In accordance with ZO-1, Claudin-1 decreased in both the OVA+Antibiotic+ group and the OVA+Antibiotic- group. As for Occludin, although the expression in the OVA+Antibiotic+ group was reduced, that in the OVA+Antibiotic- group showed no significant reduction (*p* > 0.05). The allergic reaction did not affect the expression of Occludin in intestinal epithelial cells. These results suggest that intestinal barrier injury is more severe in mice with gut microbiota dysbiosis, which may be related to the decrease in TJs expression in intestinal epithelial cells. Food allergy reduced the expression of ZO-1 and Claudin-1 in intestinal epithelial cells, but did not affect Occludin expression.

To further validate the findings, we performed immunohistochemical analyses of the TJs of mice in the gut microbiota dysbiosis model (Figure 9c,d). Likewise, gut microbiota dysbiosis reduced the expression of ZO-1, claudin-1, and Occludin in intestinal epithelial cells. Antibiotic-induced gut microbiota dysbiosis damages the TJs, leading to an increase in the OVA entering tissues through the paracellular pathway. This results in allergens that are more accessible to antigen-presenting cells, which in turn promote anaphylaxis.

Previous studies revealed that the zonulin signaling pathway could regulate intercellular TJs. Thus, we detected zonulin in mice serum. As to the food allergy–gut microbiota dysbiosis model, the mice in the OVA-Antibiotic+ group and the OVA+Antibiotic+ group showed a higher expression of zonulin (Figure 9e). Correspondingly, in the gut microbiota dysbiosis model, the zonulin content in the Intes group was significantly higher than that in the Ctrl group (Figure 9f) (*p* < 0.05). This is consistent with the results of immunohistochemistry for TJs. It is suggested that the dysbiosis of gut microbiota activates the zonulin pathway, which increases intestinal permeability, thereby aggravating food allergies.

## 4. Discussion

Previous studies have shown that food allergy is closely related to the changes in gut microbiota in early life. Through flora transplantation, it has been proven that many diseases affect food allergy through intestinal flora. Maryam transplanted the intestinal flora of obese mice fed with a high-fat diet to sterile mice, and found that the intestinal flora did not change the obesity status of the transplanted mice, but did increase the sensitivity of mice to food allergy [6].

As mentioned earlier, previous studies have analyzed the species of bacteria that affect food allergy, but the results are not consistent. There are three main reasons. First of all, in the analysis of gut microbiota, the current technology cannot analyze the changes of specific strains, and is only effective at the family and genus level. However, there may be beneficial or harmful strains in a family or a genus simultaneously, so contradictory results may appear suggesting that rumen cocci play a beneficial role in some studies, while playing a negative role in other studies. Secondly, there may be differences in the analysis systems used in these studies. Using a traditional bacterial culture often leads to a number of detectable species that is lower than expected, and the differences in the analysis systems also lead to contradictory results. Third, many of these studies are cross-sectional studies and lack continuous sampling [50,51,52,53,54].

The mechanism by which gut microbiota affect food allergies is multifaceted, and the metabolites play an important role [55]. SCFAs such as propionate and butyrate promote the health of the intestinal barrier, regulate the differentiation of Treg cells, and have an anti-allergic effect [56]. A high-fiber diet is beneficial to bacteria that can ferment dietary fiber, such as Bifidobacterium and Lactobacillus, increasing serum SCFAs level, and inhibiting food allergy [57,58]. Tryptophan metabolites, such as IPA, IA, and IAA, have been proven to represent potent mediators of the anti-inflammatory response, promoting the health of the intestinal epithelial barrier [19,28,59]. Purine metabolism is related to UA levels, and studies have shown that the abnormal purine metabolism of the intestinal flora could exacerbate colitis, revealing that it may be related to inflammation [43].

Cholera toxin (CT) is often used as an adjuvant in orally sensitized mouse models, and it is considered one of the most effective oral immunogens [60]. When sensitized after gut microbiota dysbiosis, we found that dysbiosis plays a role similar to CT adjuvant, inhibiting tolerance production. This is a supplement to previous research; the effect of antibiotic-induced gut microbiota dysbiosis in increasing susceptibility to food allergies may not only exist in early life, but may also appear later life [61,62]. Furthermore, we found an increase in TNF-α in the mice serum of this model, but the immune cells in the blood did not proliferate significantly. This mild increase in inflammation may be one reason why gut microbiota dysbiosis promotes food allergy susceptibility. We also found an increased inflammation level in the circulatory systems of mice with gut microbiota dysbiosis in the food allergy–gut microbiota dysbiosis model. Unlike the gut microbiota dysbiosis–food sensitization model, this increase in inflammatory levels, including TNF-α and blood immune cells, may be responsible for the aggravation of food allergies. This more severe inflammatory reaction also occurred in the intestinal tract. The above results indicate that gut microbiota dysbiosis aggravates the intestinal symptoms of food allergies.

In our study, gut microbiota dysbiosis reduced the level of TJs (ZO-1, Occludin, claudin-1), resulting in the damage of tight junction colonization and increased intestinal permeability. The initiation of food allergies may require disturbances in the local microenvironment in order to prevent tolerance [63]. Our research supports this point, and this may also be why gut microbiota dysbiosis caused by antibiotics increases the susceptibility and severity of food allergies. Previous studies have also verified that the house dust mite allergen Derp1 enhances the permeability of the epithelium via its proteolytic activity, reduces the expression of TJs, and weakens the mucus barrier, indicating that it is an environmental trigger of food allergy [64]. Milica M proved that the kiwi fruit allergen protein Actd1 could degrade Occludin protein and damage intestinal permeability via Caco-2 cell and animal experiments [65]. It was suggested that actd1 could decompose TJs, which is an important factor in Kiwifruit sensitization. Dwan B. demonstrated that peanut allergens destroyed the integrity of the Caco-2 barrier by impairing TJs, which allowed the allergens to pass through the intestinal epithelium via the paracellular pathway [66]. These studies confirm the role of allergen-damaged TJs in food sensitization. Food allergens are harmless. The disturbance of homeostasis in the barrier area caused by an exogenous or endogenous destructor, which activates or reduces the threshold of the immune system, is the main factor leading to an allergic reaction [67].

**Figure 10:**
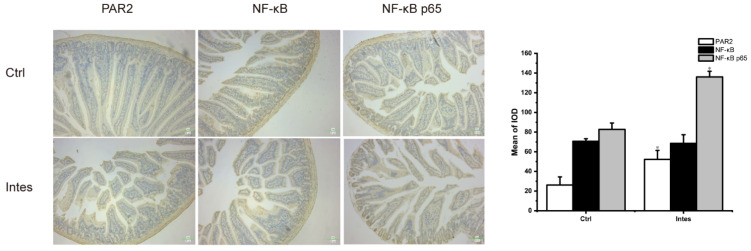
Immunohistochemical pictures and quantitative analysis of PAR2 and NF-κb in the gut microbiota dysbiosis model. * *p* < 0.05 as compared to the OVA-Antibiotic- group.

Moreover, in our study, antibiotic-induced gut microbiota dysbiosis increased the expression of protease-activated receptor 2 (PAR2) in the jejunum, and indirectly promoted the phosphorylation of NF-κB (Figure 10). PAR2 is an innate immune mediator expressed on keratinocytes, which can induce Th2-associated inflammation [68]. Recently, PAR2 has been proven to regulate TJs expression in house dust mites-mediated allergic rhinitis [69], and it can participate in the inflammatory response by activating the NF-κB pathway [70]. This finding means that antibiotic-induced gut microbiota dysbiosis may reduce the expression of TJs through the PAR2/NFκb pathway, increasing intestinal permeability and leading to an increase in serum zonulin. Nonetheless, whether the influence of antibiotic-induced gut microbiota dysbiosis on the susceptibility and severity of food allergy is also related to this signaling pathway still requires further experimental verification.

In fact, both genetic and environmental factors can affect the development of food allergies and changes in intestinal flora, which make the impact of intestinal flora on food allergy more complex. In our study, animal experiments were mainly used to simulate the gut microbiota dysbiosis of adult individuals against similar genetic backgrounds in order to understand the possible interactions. In this antibiotic-induced gut microbiota dysbiosis, we found changes in specific flora. Previous studies have shown that probiotic supplementation can avoid gut microbiota dysbiosis and prevent food allergy in infants. Our study did not address the effect of probiotic supplementation on food allergy in adults, or perform more in-depth metabolomic analysis, which remains to be studied. In addition, changes in intestinal flora not only affect the flora itself, but also affect the disease process of offspring through pregnancy and delivery. Our experiment is limited to the effects of drug-mediated intestinal flora disorders on individual food allergy. Some epidemiological studies and animal reproduction experiments have been used to discuss the effect of maternal intestinal flora on food allergy in offspring, which is also a direction worthy of further research in the future.

## 5. Conclusions

In conclusion, our study focused on the correlation between gut microbiota and food allergy in adulthood, proving that antibiotic-induced gut microbiota dysbiosis increases the susceptibility to and severity of food allergy. Structural changes of the intestinal flora were characterized by decreased beneficial bacteria, such as Lachnospiraceae and Prevotellaceae, and the growth of harmful bacteria, such as Enterococcus. Additionally, these changes affected the production of SCFAs, tryptophan metabolites, and purine metabolites, damaged intestinal barrier health, and increased the susceptibility to and severity of food allergy. Gut microbiota dysbiosis could play a role similar to CT adjuvant in establishing a food allergy model and avoiding tolerance in mice. The reason for this is that gut microbiota dysbiosis produced systemic and local inflammation, which led to intestinal barrier damage. The dysbiosis reduced TJs, increased zonulin in serum, and aggravated the symptoms of food allergies. Further studies are needed to explore the signal mechanism of the effect of antibiotic-induced gut microbiota dysbiosis on the susceptibility to and severity of food allergies, and the specific mechanism by which the structure of the intestinal flora and intestinal metabolites affects the susceptibility to and severity of food allergy.

## Data Availability

The data supporting the findings reported herein are available on request, from the corresponding author.
